# The Virus Covid-19 and Dilemmas of Online Technology

**DOI:** 10.17650/2712-7672-2020-1-2-64-71

**Published:** 2020-12-04

**Authors:** Roger Smith

**Affiliations:** Lancaster University; Institute of Philosophy of the Russian Academy of Sciences

**Keywords:** COVID-19, online communication, technological change, touch, movement, COVID-19, общение онлайн, технологические изменения, прикосновение, движение

## Abstract

Commentary on the COVID-19 pandemic must necessarily consider the medical issues in social and political context. This paper discusses one important dimension of the context, the long-term history of human activity as intrinsically technological in its nature. The pandemic has accelerated the use of technology to mediate relations between people at a distance. This involves not only a change in the skills people have (though acquiring these skills has become the central project of work for many people), but changes the sort of person they are. Our notions of closeness and distance, or of touching and being touched, and so on, refer simultaneously to states that are spatial and emotional, factual and evaluative. Inquiry into the differences in human relations where there is physical presence and where there is not raises very significant questions. What are the differences and why are they thought, and felt, to matter? What are the differences when the relationship is supposed to be a therapeutic one? What are the financial and political interests at work in enforcing relations at a distance by new media, i.e., mediated relations? How is a persons agency affected by a lack of freedom to move or a lack of face-to-face contact? What happens to all those human relations for which physical presence was previously the norm, relations such as those performed in the rituals of birth, marriage and death, or in activities like sport and the arts? Can it be said that new technologies involve a loss of soul? The present paper seeks to provide a reflective and open-ended framework for asking such questions.

## "NATURAL" HUMAN TECHNOLOGY

There are clouds of commentary, and not a little fog, on the medical dimensions of the impact of the virus Covid-19. Factually grounded surveys of people's reactions and fears, and calls for appropriate online responses, are therefore most welcome. An appreciation of the wider historical and social setting is, however, still needed. The issues are very complex, and in this commentary I focus on the restrictions to people's movement and the rapid shift to online rather than face- to-face encounters. I discuss the shift to technologically- mediated communication. It is true that, for many millions of individuals, digital encounters were a daily, sometimes almost continuous, reality or norm, long before the pandemic. Earlier, however, personal face- to-face meetings accompanied the digital reality, and face-to-face meetings formed a default position or baseline with reference to which people experienced and assessed relations at a distance. Moreover, many areas of everyday activity, including work, childcare, education, rituals of the life cycle, entertainment, conversation, sport and the arts, centred on people physically coming together. During the pandemic, relations at a distance suddenly, literally overnight, became the new norm, enforced by police powers and not freely chosen. In this commentary, I raise existential, as opposed to specifically medical, questions about what happens when people do not physically come together and are not allowed physically to come together, but instead relate via technology.

Humans are by nature instrumental in activity; human beings do not exist and then add technologies to what they do, rather they use technologies to enlarge the capacities they have in their nature: "We are designers by nature"[1]. It is wrong to discuss technology merely as a means, available to be used or misused; technology is not "added on" but "given in" the very notion of what it is to be human. Technology is the form of human self-making.

We therefore cannot say that the shift to online relations is unnatural. However, we can ask whether the technological innovation is on such a scale that it marks a break or revolution in history. Observers in other ages reported a sense of overwhelming novelty of the technological change, for example, in response to the speed of movement and the shrinking of distance, with the advent of railway transport. It is commonplace today to refer to the "revolutionary" transformations underway as a result of new biomedical and digital technologies. It is a judgment which follows from the belief that new technologies alter the very nature of being a person by rebuilding or re-engineering the body, whether through genetic manipulation, prostheses, cyborg systems, drugs, surgery, or in some other way. There is similar talk about the "revolutionary" replacement of reality with electronic virtual realities. There is discourse about "the transhuman"; some people judge that the category "human" is so closely associated with beliefs about the fixed character of universal, basic and natural human qualities or capacities, that given the changes that technology now makes possible, it is of no further use. With all this acknowledged, though, it is not clear that contemporary changes are completely novel. We must also question whether we can distinguish what is new from people's experience of what is new.

New technologies appear to challenge the presumption that human nature stays the same, that all people have had and will continue to have large areas of capacity and character in common. Stanistaw Lem, half a century ago, accurately foresaw the social event now taking place, the event questioning the very notion of a fixed human nature: "Man remains the last relic of Nature, the last 'authentic product of Nature' inside the world he is creating. This state of events cannot last for an indefinite period of time. The invasion of technology created by man into his body is inevitable"[2]. When people use new communications technology, the technology appears to substitute for the body. Yet this could also be said about the invention of the technology of the wheel, or of the stirrup, technology which enabled people to be carried rather than having to walk.

With these general points in view, it is possible more clearly to formulate the question people are asking: What changes are occurring with the switch, imposed by government controls, from physical encounters to digital encounters between people? We cannot think that people will remain exactly the same when they adapt to physical isolation technologies. I am not making the obvious point that people will have different habits and different skills, though they will, rather I am suggesting That, acquiring these habits and skills changes the sort of person they are mentally, morally, or if you will, spiritually, as well as materially. At the level of detail, the changes vary enormously from society to society, group to group, situation to situation and individual to individual. It is an empirical matter to study this, as surveys do, while each of us can contribute impressionistic knowledge. I am now suggesting a framework to enable the creation of a general picture.

## PHYSICAL PRESENCE AND THE MEDIATION OF RELATIONS

These general points enable us to avoid being trapped by questions about the newness or revolutionary nature of present technologies, or about whether they have entirely new evaluative dimensions. The new technologies and circumstances of isolation pose no absolutely new dilemmas. However, they do give people new experiences and pose social, medical and political choices in particular terms.

In all media of communication, including verbal messages, signalling, letters, books, the telegraph, the telephone, radio and TV, a person may have relations with other people who are not physically present, not within touchable range. Individual reactions to working and communicating via contemporary media vary enormously, just as earlier reactions varied. These reactions are laden with values and emotion. Whenever we talk about closeness and distance and "being in touch" we are at one and the same time talking in spatial, emotional and evaluative terms. This is how ordinary language works. Technological change cannot but affect emotional and evaluative worlds.

Training and habit are certainly factors in the way people feel the closeness or distance of others when using different media. These factors may be so deeply embedded that they feel natural, though the experience of what is natural is also cultural. We do, as matter of fact, in many circumstances, but not all, value the physical presence of others. There are undoubtedly special emotional pleasures and pains associated with this. It has been reported that even teenagers devoted to continuous online relations with friends, in conditions of lockdown began to miss physical presence. Even these people, the most habituated to relations at a distance, find something different in presence. Why is this?

Humans are embodied subjects. Thus, it can be said that if a human is truly present, an embodied subject is present. Online, therefore, "the whole person" is not there. "Presence" is the here and now of embodiment. We may imagine a culture in which the presence of the embodied subject does not matter, but this would not be a human culture as currently understood. Many people will think the physical closeness of mother and new-born child as "natural" because the child is at first embodied in the mother. An influential body of psychoanalytic thought (in the Kleinian tradition) maintains that the qualitative character of literal contact between mother and child, perhaps already in the foetal state, and certainly with contact through lips and breast after birth, determines all subsequent forms of relations. The child does not feed at the breast by Zoom. If a medium, like a bottle, mediates lips and breasts, or if a mother is absent, it follows that the child's psyche develops differently.

It is from being embodied and present that people can "touch" and "move" another person, and themselves "be touched" and "moved". This is language which simultaneously describes the moral, or spiritual, and physical dimensions of life. (It is not a matter of metaphor running from physical to mental worlds, or vice versa, but of commonality of meaning.) The rich content or resonance of such language is lost online. Wholeness in touching and moving, and it may be argued also in healing, requires the embodied person. The importance of touch is widely recognized. It features in a large survey recently carried out in the UK [3]. It is also evident in the large financial investment IT corporations are making in developing tactile media, turning tactile reality into marketable commodities which will not actually require presence. In many circumstances, what we think of as good relations involve physical contact, are three-dimensional, implicate and respond to the whole embodied person, and involve continuous movement. Think, for example, of the friendship and trust embodied in the handshake, let alone the kiss or placing the ring on the finger.

With physical distance, there certainly can be relationships, but they are different. With modern technology, though relations may still be visually face- to-face, the faces are two-dimensional, more like masks than embodied faces. The people communicating are not in three-dimensional moving spatial relations. There is no touch, and there is no "con-tact". Though in ordinary speech we talk about "getting in touch" and "getting in contact" by email, by phone or by Skype, taken literally this is precisely what all existing communication media do not permit: they do not permit touch or contact. There are many situations in which relations at a distance are much desired, for example, regulating the spread of a virus. However, there are many other situations, such as the relationship between mother and child, or in caring for an ill person, where the opposite is the case. In English, people talk about the importance of "hands-on" experience.

Intimate relations are by no means the only situations in which actual physical presence is thought to be of decisive importance. For example, people have fought wars over claims about the literal physical presence of the blood of Christ in the Christian mass, as opposed to the symbolic presence. Or, consider the wish that people have physically to attend marriages or funerals, though that attendance is symbolic. In the arts there is a wish to attend live performances and not only to watch or listen to a recorded performance or a performance specially created for a digital medium (such as pop music or dance videos). Similarly, people wish to attend football matches, or travel, even though they can see much more, much more clearly, on the screen at home. This kind of argument is not limited to the world of the arts or sport. There is, for instance, a direct parallel in the experience of landscape, in the contrast between moving in a landscape while walking, and in gazing as a visitor at a landscape [4].

The concept of presence is valuable, as it makes possible an understanding of the difference between a live performance and a recording, of walking in a landscape as opposed to watching a travel film, or of dancing as opposed to watching a dance video. The concept of presence is central to discussions around performance aesthetics; it is also of great importance to healing.

For people in the culture in which I write, concern with existence inescapably involves inquiry into the embodied subject, that is, physical presence. "Being close" or "being distant", "moving" or "being moved", is the source of the very notion of significance, of something mattering. How individual people feel varies, but it is the mattering that is the source of the qualities of physical face-to-face encounters. The value that comes with being alive rather than dead, requires the feel of something in relation to something else [5,6]. Things and events have value to people because something in the world "pushes back", something offers resistance. The moving body knows such resistance from the earliest moments of sensory consciousness. This may be expressed in abstract philosophical terms or in concrete psychological terms, recognizing the primary significance of kinaesthetic sensation in the subjective world.

All this confirms "the obvious": in the societies in which we actually live, with the forms of life we have, as a matter of fact people place a great deal of weight on physical presence. The question is whether this is changing as a response to the virus and if it is, what are the consequences of people reducing or diminishing their desire to act with other people who are physically present? In brief, what changes are happening and in what sense can we say they matter? To answer these questions require attention to the social and political implications of closeness and distance, and of the technology that mediates relations at a distance.

## AGENCY AND THE TECHNOLOGY OF MEDIA

Particular experiences and practices are infinitely varied, they are highly individual and they are not entirely rule-bound. These are characteristics that machines find difficult to reproduce. This is the subject matter of a film inspired by Herbert Dreyfus, the early proponent of the belief that artificial intelligence machines will not reproduce human actions because human actions involve innovation and risk [7]. The distinctive characteristics of individual practices, or of the practices of the group to which people belong, are felt to be part of what matters about the practices. The imposition of new technologies, whether by commercial pressure or police powers, may threaten or even eliminate such distinctiveness. If the same technologies mediate all relations, the value that any particular relation has, the mattering it has to a person, becomes homogenized, flattened. (There is a logical point at issue here: if there was no differentiation of values there would be no value at all - quality depends on difference.) Value is value to me, and it cannot be dissociated from my embodied difference. If the only permissible route to doing something is the route that all others must take, there is no longer anything of value to defend. This is the dystopian dream of pure instrumentality, everyone doing the same thing through the same technology to achieve the same outcome. It is the power of media technologies to move society in this direction. However, new technologies may at the same time offer new possibilities in practice to those with access to, and mastery of, the technologies.

As the word "media" indicates, contact through a medium, i.e., contact at a distance, creates a space or "medium" where forces or powers are at work other than the will and reason of the communicating people, powers that are embedded in the medium. This is the case for all communication technologies, beginning with gesture and language, and it is emphatically the case for digital technologies. Corporations design and manufacture these technologies, and institutions and governments regulate their use, establishing vast zones in which individuals do not have the power of decision. Media mediate political and financial powers. Any judgment about the effects of a switch to online communication must therefore take into account the relations of individual or local agency and the technological agency mediating corporate and political powers. To say this is to say nothing new; there is a considerable amount of discussion of these issues in media and cultural studies. If we think of a person as a locus of individual agency, and if the agency of that person is mediated online, then we have to consider the person as a person in whom a range of social powers is at work. This is how one Greek correspondent, a businesswoman, expressed her new experience of work during lockdown (personal email, June 21, 2020): "[it] is not a different depiction of reality, not even a different, technologically mediated interaction with reality; it is a different reality altogether, one which is not bound by the rules we, collectively, had negotiated and agreed upon or at least consented to. To exchange one for the other... is to accept a different social contract without even been given the opportunity to understand its implications. From face recognition, to internet trade, to the abolishment of the physical workplace, our immediate understanding of our own life, of our individuality, of our connection to others, is vanishing - to be substituted with what?"

Questions of spatial distance are thus inseparable from questions of agency and power. Everything that exists is in relations - from electrons to people. In stating knowledge of relations, we map an understanding of cause and effect and describe where the power lies to cause something to happen. Being face-to-face with someone, we recognize a person's capacity to influence another person, either directly through physical coercion or caress, or indirectly through speech, emotional display or gesture. This capacity is more difficult to recognize in contact mediated by a technology, since this involves assessing the influence that the technology itself has in the field of relations. Behind the technology is the long and complex history of the workings of powers that have produced the technology and made it available under certain conditions. It is striking and often painful that, for many users of modern digital media, especially for an older generation for whom these skills are harder to acquire, it is the technology that determines the range of options, rather than the will of the person using the technology.

There is deep ambivalence in people's responses. Digital technology empowers people by making communication possible where otherwise it would be impossible. Some people, especially children growing up with the technology, feel this creates opportunity. At the same time, these technologies introduce into communicative relationships a large raft of powers to which the people in communication have to conform, and this disempowers individual people understood as agents. The emotional distance, and in many cases alienation, that many people feel when "in touch" by digital media, is an expression of this disempowerment. As teachers adapt to teaching online, who is empowered, who disempowered?

Policies to contain the virus require people to go online rather than use transport and public buildings or public spaces in order to work and to meet. Such policies have narrowed the range of choice of communication, requiring people to adopt a restricted range of technologies and, effectively to use those technologies to conform to practices imposed by police powers or by employers. Some people have quickly become comfortable with new conference or teaching technology, others have not. To become comfortable, people study and train as users of the technology, engage in activities different from the activities which previously defined their occupational identity. Online conferencing illustrates a common phenomenon in the spread of IT culture in general: the technology shapes the time and expertise that people have. The massive advantages this has for management and bureaucracy, bringing diverse activities under a common description, and hence common possibilities for planning and assessment, is all too clear in universities. There is a massive transfer of time, effort and commitment to mastering IT technologies rather than using competencies for activities not so easily planned and assessed. The same process is evident at the level of national governance, in health services, and so on.

## CONCLUSION: THE RECREATION OF RELATIONS

All this would seem to confirm the characterization of modernity presented, for example, by the philosopher Heidegger: the contemporary age has an instrumental understanding of Being - all actions and judgments are subsumed by the value of technical efficiency [8]. Observers more sensitive to politics than Heidegger would add that this very much serves the interests of capital and authoritarian government. The capacity to transfer relationships online and recreate relationships in terms of instrumental understanding, is powerful indeed.

The recreating of relationships, this commentary argues, involves narrowing the range of responsiveness. With a person physically present, all the bodily senses are at work. Movement, even if slight, gives the person three dimensions, and there are much greater possibilities for flexibility, subtlety and articulacy in communication because of the involvement of the whole body. Noise introduced by the technology is absent. (By noise, I mean sound or other data which are not part of the communicative act.) It is a large question as to whether there is a lessening of emotional concern online, a tendency towards affectively relating to people as technologically constructed objects rather than subjects. This links with what I am saying regarding the gaze, the constitution of identity and power relations through visual presentation, the look that each person presents to others, and the relationship of each performance artist with their audience. Here again, there is a great deal of literature, much informed by ethnic and gender identity issues. I suggest that perception in two- rather than three-dimensional terms is not only visually different but evaluatively different. In the moral sphere, to recognize three-dimensionality is to recognize complexity, to attribute to the subject of the gaze ("the other"), a richness that a two-dimensional representation does not have. The novel is a paradigm of the threedimensional form of representation, the advertisement a paradigm of the two-dimensional form. Online relations appear to encourage familiarity with and acceptance of two-dimensional moral relations. This is reduced to parody in recording "like" or "dislike" using the up-turned or down-turned line of the mouth, the two-dimensions that the machine recognizes, emotions reducible to digital relations, on or off, black or white, them or us.

The opposite condition to this technological distancing is the state of literally being "in touch", that is, touching. Touching is the human relationship in which the life- worlds of individuals most materially flow together and each person has the status of a multi-dimensional subject. For this reason, we have large experience and training in learning when to touch and when not to touch. I would add that touching, whether active or passive touching, involves movement, and that the sense or touch is always informed by bodily senses and by the kinaesthetic sense [5]. At an online conference, these senses are narrowed down to mild physical discomfort sitting before a screen on which people in awkward poses speak in a mainly monological manner. Of course, with practice, the online experience may improve. However, if forced to go online, I have to learn to shift my freely chosen way of life and to restrict my mobility.

Physical presence matters not just because it is a habit, but for what we may call existential as well as for moral and political reasons. Lem provided an analogy which helps us to understand this. Discussing the difference between an authentic and a forged painting when only an expert can tell the difference (and indeed when even experts may have different opinions), he wrote: "[The forgery] is empirically indistinguishable from the original, but is not the original, as it has a different history" [3]. Analogously, the difference between reality and virtual reality, or between physical presence and contact at a distance, is that these states have different histories; they occupy different positions in the stories with which we give meaning to ourselves and the world. Removing the possibility of physical closeness removes the stories, with all their meaning, that we might want to tell about being significant subjects in meaningful relations. We have to learn to tell new stories - stories imposed on us and, as such, carrying a different meaning. This is a very significant argument. Reality and virtual reality differ for us because they bear a different meaning given by their different histories. An online course of education does not have the same history as a course with teacher and student physically present with each other. Online medical advice is part of a story that differs from "hands-on" contact.

I am tempted to use the language of "loss of soul" in relation to the shift to online practices. This is risky, because the language of soul is open to considerable vagueness and misunderstanding. If I turn to this language, I borrow from what I understand of the world of black slaves in the formation of African- American culture, the culture which spread the reference to soul in modern secular English. Slave culture used the word "soul" to refer to the individual quality of a person, imagined in ideal terms, the quality that is not and cannot be the property of another person. The soul, understood in this way, can be killed or destroyed, or possessed by an alien spirit, but it cannot be bought or sold. If people "sell their soul", the soul is lost. By analogy, if something is said to have soul, it is said to have value that cannot be exchanged for something else (especially money). Thus used, reference to soul does not refer to an "I" but to a condition of being in relationship to value. To be "soul-less" is to be in a place or time where no such value is possible or imaginable. This language, I want to make explicit, does not argue for the soul as a metaphysical or transcendent entity but refers to a culturally embedded conception that certain kinds of human relations, existing in particular social times and places, cannot be exchanged. Such conceptions are individually and collectively central to many people's sense of purpose and identity.

IT technologies narrow down the scope of soul as a viable category of self-understanding. Large social institutions, especially corporations and governments, have an interest in narrowing the scope of soul, because it is by definition outside of ownership and regulation. Thus, the fear is that policies of lockdown hurt the soul. Face-to-face encounter has the potential to restore the scope of the language of the soul.

It is the thrust of this essay that technologies change ways of life and forms of being human. The transformation of media technologies was well underway before the Covid-19 pandemic, but the virus very much speeded it up. The rate of change has been central to the way people have reacted. The sudden spread of the new technologies in conditions of isolation has forced even conservative users of technology to question any conventional separation between natural and technologically mediated activity. Policies imposed in response to the virus have accelerated a pattern of change, rather than imposed anything new, bringing more people, more quickly, under new regimes of management and governance, making everybody, and not just those already fully engaged with online relations, face the relativity of the natural/ technological distinction.

The sudden switch to online relations has dislocated many people's everyday ways of sustaining relations and meaning. There has been damage to feelings of selfagency and identity that heretofore depended on direct contact with other people. In these circumstances the resulting change in attribution of agency, or causal power, has varied hugely with individual, social, economic and political context. Nevertheless, it is surely right to say that the pandemic very rapidly led governments to enhance the agency of the technology, and through the technology enhance the agency of those social institutions which can use the technology for their own ends. These institutions are, firstly, governments themselves, secondly, the corporations that design and supply the technologies, and thirdly the institutions like universities or health services that use the technologies to take greater control of the lives of the people they employ and, in principle, serve.

The internet is a technology for creating relations "out there", in space, or hyperspace, though this too is a social space. People who live on the internet live differently from people who do not; literally, their identity is different. If, because of the virus, or because of the attractiveness of new powers of technologically-mediated governance, we are all forced online for everything, then the identity of being a person changes. Life has a different history and a different meaning.

## Acknowledgements

The author gratefully acknowledges the comments of Ruth O'Dowd, Irina Sirotkina, Maria Yamiladou and participants in discussions of the class in English of the Institute of Philosophy, Moscow, led by Olga Zubets.

## Funding

The author declares no financial support in the preparation of the article.

## Conflict of interest

The author declares no conflict of interest in the preparation of the article.
